# The Western Diet and Atopic Dermatitis: The Potential Role of Nutrients, Contaminants, and Additives in Dysbiosis and Epithelial Barrier Dysfunction

**DOI:** 10.3390/antiox14040386

**Published:** 2025-03-25

**Authors:** Chiara Maria Teresa Boggio, Federica Veronese, Marta Armari, Elisa Zavattaro, Elia Esposto, Paola Savoia, Barbara Azzimonti

**Affiliations:** 1 Laboratory of Applied Microbiology, Department of Health Sciences (DiSS), Center for Translational Research on Allergic and Autoimmune Diseases (CAAD), School of Medicine, Università del Piemonte Orientale (UPO), Corso Trieste 15/A, 28100 Novara, Italy; chiara.boggio@uniupo.it (C.M.T.B.); marta.armari@uniupo.it (M.A.); barbara.azzimonti@med.uniupo.it (B.A.); 2 Dermatology Unit, Department of Health Sciences (DiSS), School of Medicine, Università del Piemonte Orientale (UPO), Via Solaroli 17, 28100 Novara, Italy; federica.veronese@med.uniupo.it (F.V.); elisa.zavattaro@med.uniupo.it (E.Z.); espostoelia@gmail.com (E.E.)

**Keywords:** atopic dermatitis, skin microbiota, *Staphylococcus aureus*, exposome, Western diet, dysbiosis

## Abstract

Atopic dermatitis (AD) is a chronic inflammatory skin disorder influenced by both genetic and environmental factors, collectively termed the exposome. Among these determinants, diet emerges as a pivotal component, with diverse nutrients, contaminants, and additives shaping immune responses, microbiota composition, and systemic inflammatory status. This literature review aimed to elucidate the interplay between dietary factors and skin dysbiosis in AD, providing insights into how these interactions may impact disease susceptibility and progression. A comprehensive search of PubMed and Scopus was conducted using relevant keywords and medical subject headings (MeSH). Studies published in English within the past 25 years were included, encompassing in vitro, in vivo, and ex vivo research, as well as reviews. Priority was given to frequently cited articles, reflecting significant contributions to current understanding. Findings suggest that dietary habits influence AD by modulating both gut and skin microbiota, immune pathways, and inflammatory processes. These insights underscore the importance of considering diet within a broader exposome framework, paving the way for targeted interventions to improve AD management. Further research is needed to clarify the mechanisms and optimize nutritional strategies, potentially informing preventive and therapeutic approaches for AD.

## 1. Introduction

The exposome is a comprehensive concept that represents the totality of environmental exposures that an individual encounters from conception onward throughout their lifetime [1]. It encompasses all non-genetic factors that can influence well-being, interacting with genetic predispositions to shape physiological responses and health outcomes. Dietary habits represent a significant part of the exposome and can have a significant influence on various health conditions, including inflammatory and immune-related disorders such as atopic dermatitis (AD). In this context, diet interacts with other exposome elements, influencing skin health, eubiosis, immune responses, inflammatory status, and overall susceptibility to AD [1].

In this literature review, a comprehensive search was conducted using the electronic databases PubMed and Scopus, which are both publicly accessible and renowned for their extensive coverage of the biomedical literature. A combination of keywords and medical subject headings (MeSH) was employed to ensure a thorough retrieval of relevant studies. The search terms included: “inflammatory skin diseases”, “atopic dermatitis”, “atopic eczema”, “exposome”, “diet”, “western diet”, “mediterranean diet”, “nutrients”, “contaminants”, “additives”, “epithelial barrier”, “skin microbiota”, and “skin microbiome”. These terms were used both individually and in combination to capture a wide array of the pertinent literature. The focus was on original, peer-reviewed scientific studies encompassing in vitro, in vivo, and ex vivo research, as well as comprehensive reviews. High-quality, indexed articles that have been frequently cited, indicating their impact and relevance in the field, were prioritized. The selection criteria were confined to international studies published in English within the past 25 years, ensuring the inclusion of contemporary research while acknowledging foundational studies that have significantly contributed to current understanding. Globally, 148 papers were identified, providing a comprehensive and up-to-date overview of the external factors influencing AD.

The aim was to comprehensively examine the interplay between the exposome, particularly dietary habits, and skin dysbiosis in the context of AD. We seek to elucidate how various dietary factors—including nutrients, contaminants, and additives—interact with environmental exposures to influence the pathogenesis and progression of AD, providing a nuanced understanding of these interactions and thereby informing potential nutritional and environmental interventions for the prevention and management of AD.

## 2. The Exposome and Human Microbiota

The exposome, which encompasses the totality of internal (endogenous processes such as hormonal fluctuations), specific external (e.g., UV radiation, pollutants, allergens, and processed food), and general external factors (e.g., climate and socioeconomic conditions) experienced by each living organism throughout its lifetime, highly influences genetic predisposition to diseases, thus shaping health outcomes [2].

Although it is generally recognized that the possibility of developing certain chronic diseases is equally determined by both genetic and environmental factors, recent evidence suggests that around 70–90% of the total risk is instead due to differences in environmental exposure, contributing to disease progression [3,4,5]. As described by Rappaport and Smith in 2010, “*toxic effects are mediated through chemicals that alter critical molecules, cells, and physiological processes inside the body. […] Under this view, exposures are not restricted to chemicals (toxicants) entering the body from air, water, or food, for example, but also include chemicals produced by inflammation, oxidative stress, lipid peroxidation, infections, gut flora, and other natural processes*” [6]. In the last 15 years, researchers have highlighted how environmental factors and lifestyle choices can significantly impact the host microbiota, the complex community of microorganisms living in and on the human body, shaping its composition, variety, and function [7]. Indeed, all these stressors can drive the transition from eubiosis to dysbiosis, which is known to be involved in the etiopathogenesis and/or progression of a wide range of health issues [7].

### Role of Diet in Microbiota Modulation

Diet is one of the most significant modulators of the human microbiota, influencing its composition, diversity, and function. Balanced diets promote a diverse and resilient microbiota, which is critical for maintaining health. Poor diets possess low nutritional quality and lack prebiotic fibers, which are essential for the growth of beneficial microbes, thus leading to gut dysbiosis and systemic inflammation and potentially contributing to skin conditions such as acne, AD, and psoriasis through the so-called gut–skin axis [8].

A Western-style diet (WD), characterized by a high intake of processed foods, refined sugars, and unhealthy fats, as well as a low amount of fiber, has profound effects on both gut and skin microbiota. Foods rich in fat, simple and complex sugars, and salt decrease the diversity of gut bacteria, favoring the growth and virulence of potentially pathogenic species [9]. As evidenced by several studies, consumption of a WD leads to dysregulated microbiota profiles not only in mice but also in humans, with an increased *Bacillota* (formerly *Firmicutes*)-to-*Bacteroidetes* ratio, while transitioning from a WD to a control diet (CD) reduces this ratio and alleviates inflammation [10,11,12]. A WD is associated with increased inflammatory cytokine production and gut permeability, also known as “leaky gut”. As a result, it allows microbial metabolites and lipopolysaccharides (LPSs) to enter the bloodstream, thus promoting systemic inflammation [13]. Moreover, reduced fiber intake deprives beneficial gut microbes of the substrates necessary to produce short-chain fatty acids (SCFAs), such as propionate, acetate, and butyrate, which help maintain skin barrier integrity and health while reducing inflammation [14]. Lastly, processed foods can often contain additives, preservatives, heavy metals, and emulsifiers that can negatively impact host microbiota. For instance, certain emulsifiers have been shown to alter gut microbial communities and promote inflammation [15]. In particular, as demonstrated by Nielsen and colleagues in 2018, the non-ionic Tween 80 increases both the growth rate of planktonic *Staphylococcus aureus* and its total biofilm biomass in vitro.

## 3. The Exposome and AD

As with other inflammatory autoimmune skin syndromes, the prevalence of AD has increased worldwide. In the last few decades, it has more than doubled in industrialized countries, with percentages hovering around 30% and 10%, respectively, in children and adults [16]. Although it often disappears on its own with time, this condition can flare up periodically; otherwise, it can persist without any possibility of predicting its progression.

Indeed, despite all the knowledge acquired about this disease, much is still unknown regarding the causative agents. More recently, most efforts have focused on the environmental exposome that, together with genetic predisposition and/or innate immunity defects, may be associated with an increased risk of AD onset and progression [17]. Above all, processed food and eating habits seem to have a non-negligible role, even if this is mostly unclear and full of contradictions [18,19].

### Features of the Epithelial Barrier in AD

A central feature of AD is skin barrier dysfunction, which plays a pivotal role in disease pathogenesis. The epidermal barrier, primarily located in the outermost layer of the skin called the stratum corneum, serves as a protective shield against environmental aggressors such as pathogens, allergens, and irritants. It also prevents excessive water loss, maintaining skin hydration and overall health. This barrier is composed of corneocytes (i.e., dead skin cells) embedded in a hydrolipidic matrix, described metaphorically as “bricks and mortar”, where corneocytes are the bricks and lipids are the mortar [20].

In individuals affected by AD, several factors contribute to skin barrier impairment (Table 1):

(i) Filaggrin Deficiency: Filaggrin is a crucial protein involved in skin barrier formation. Loss-of-function mutations in the filaggrin gene are frequent in AD patients and can lead to a compromised barrier, increasing susceptibility to irritants and allergens. Additionally, these mutations are recognized as risk factors for bacterial and viral skin infections [21].
(ii) Altered Lipid Composition: The lipid matrix in the stratum corneum is essential for barrier function. In AD, there is often a reduction in the abundance of ceramides and other lipids, leading to increased transepidermal water loss (TEWL) and skin dryness [22,23]. This dysfunction contributes to a cycle of inflammation and pruritus, significantly impacting the patient’s quality of life.
(iii) Immune Dysregulation: AD is characterized by chronic inflammation, with a predominant Th2 response during acute phases and a shift towards Th1 dominance in chronic stages. This overactive immune response disrupts the skin barrier, perpetuating a cycle of irritation and inflammation [24,25].

**Table 1:** Factors contributing to the development of AD.

**GENETICS**	Loss-of-function mutations in the filaggrin gene [21]	Compromised skin barrier. Increased susceptibility to irritants and allergens. Viral and bacterial infection.
**SKIN** **FEATURES**	Lower abundances of ceramides [22,23]	Increased TEWL. Skin dryness. Pruritus.
**IMMUNE** **SYSTEM**	Th1/Th2 imbalance [24,25]	Compromised skin barrier. Inflammation.
**MICROBIOTA**	Skin and gut dysbiosis [26,27]	Bacterial colonization. Inflammation.
**ENVIRONMENT**	Pollutants Temperature Humidity	Compromised skin barrier. Skin dryness. Irritation.

This skin barrier impairment facilitates the entry of allergens, triggering immune responses and exacerbating inflammation, and is associated with skin dysbiosis, which is characterized by an overabundance of *S. aureus*. *S. aureus* colonization is prevalent in over 90% of AD patients, thus worsening their inflammatory status and contributing to the perpetuation of AD manifestations [17]. Another AD hallmark is reduced microbial diversity, which concerns not only bacteria but also fungi (e.g., *Malassezia* spp.). Viruses are involved as well, as data regarding a change in the profile of bacteriophages in AD have been reported [26]. Besides these changes in microbiota composition, local dysbiosis also facilitates colonization by opportunistic bacteria (e.g., the previously mentioned *S. aureus*) and viruses such as herpes simplex (HSV-1) and varicella zoster (HHV-3), which can worsen AD symptoms [27].

**Figure 1:**
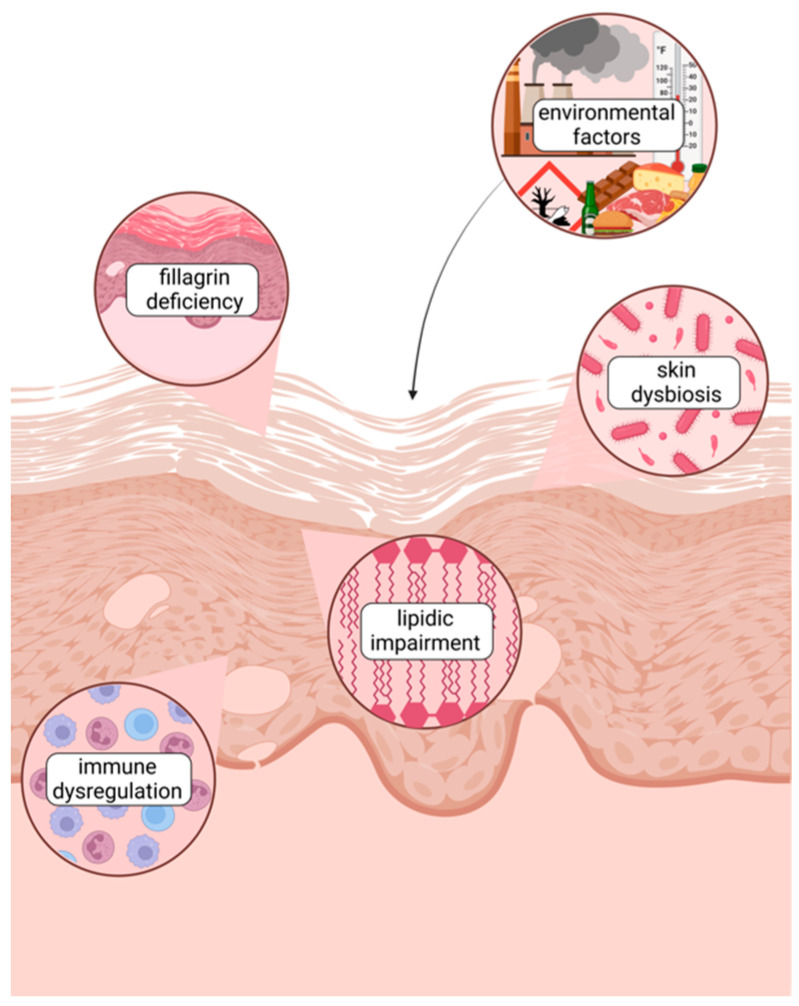
Factors contributing to skin barrier dysfunction in AD. Created with Biorender.com.

Even more important is the role of an impaired skin barrier in increasing sensitivity to environmental factors, triggering frequent flare-ups of the disease. Exposure to environmental pollutants, such as fine particulate matter from vehicle emissions and industrial activities and the gaseous pollutants (NO_2_ and SO_2_) commonly found in urban environments, as well as seasonal climate variations, is indeed associated with the worsening of AD symptoms [27,28]. Extremes in temperature and humidity can also adversely affect the skin barrier. Low humidity and cold temperatures often lead to increased skin dryness and irritation, whereas high humidity and heat can induce sweating, which may irritate the skin and trigger flare-ups [29] (Figure 1).

Consequently, strategies to preserve and strengthen the skin barrier are central to managing this condition; by focusing on local (topical) measures—regular moisturizing, gentle cleansing, and steering clear of known irritants—individuals with AD can significantly reduce skin dryness and irritation, helping them maintain healthier skin and improve their quality of life.

## 4. Role of the WD in AD

### 4.1. Features of a WD

In recent decades, the prevalence of the standard American Western diet (SAWD, here referred to as just WD) has increased globally [30]. The resulting high intake of processed, prepackaged food (commonly referred to as “junk food”), which is often calorie-dense and nutrient-poor, red and processed meats, sugars, saturated fats, and refined grains, along with the insufficient consumption of fruits, vegetables, whole grains, nuts, and seeds, lead to low dietary fiber intake and potential deficiencies in essential vitamins and minerals [31,32]. On the contrary, a Mediterranean diet (MD) is characterized by a high intake of fruits, vegetables, nuts, whole grains, fish, poultry, and dairy products [33].

**Figure 2:**
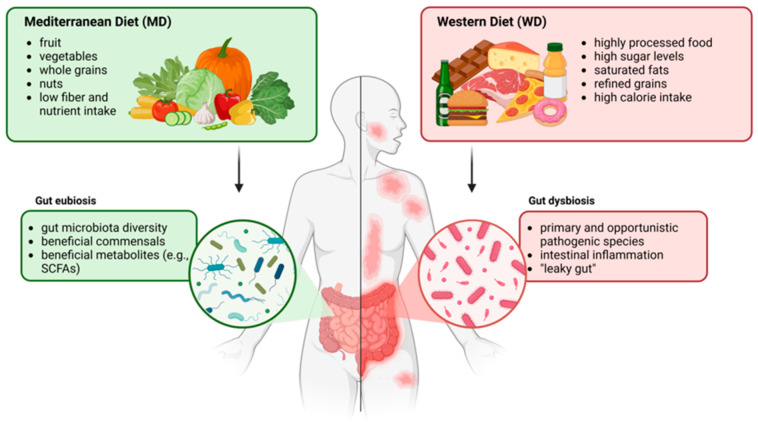
Comparison between the Mediterranean Diet (MD, on the left) and the Western Diet (WD, on the right) and their effects on the gut microbiota. Created with Biorender.com.

A WD dietary pattern is associated with increased risks of chronic diseases, including obesity, type 2 diabetes, cardiovascular diseases, and certain cancers [30,31,32,33,34]. In contrast, the polyphenols contained in the plant-based foods typical of the MD have a range of health benefits, including anti-inflammatory, antioxidant, neuroprotective, and anti-cancer properties [35,36]. Both the MD and WD impact the gut microbiota, supporting eubiosis and promoting dysbiosis, respectively (Figure 2) [37].

### 4.2. Role of the Gut–Skin Axis and Microbiota

A healthy and diverse gut microbiota, supported by a fiber-rich diet with prebiotics and probiotics, can support immune function and potentially mitigate the inflammatory responses associated with AD. Conversely, gut dysbiosis—characterized by an imbalance in or a loss of microbial diversity—is linked to elevated inflammatory markers and may negatively affect skin health. The recently introduced “epithelial barrier hypothesis” provides insight into how these factors influence overall health. This hypothesis suggests that the disruption of cutaneous, respiratory, and gastrointestinal barriers, often driven by reduced microbiota biodiversity, allows the overgrowth and increased virulence of pathobionts. In turn, this alteration promotes local and systemic inflammatory processes due to the penetration of bacteria into the bloodstream, exacerbating disease states [17].

The gut–skin axis refers to the intricate bidirectional communication between the gastrointestinal system and the skin and is mediated by the gut and skin microbiota, the immune system, and metabolic pathways [38]. This connection highlights how gut health and microbial balance can influence skin health and vice versa through a continuous feedback loop. The gut harbors trillions of microorganisms, including bacteria, fungi, and viruses, that perform critical functions such as nutrient metabolism, immune modulation, and the production of bioactive compounds such as SCFAs. When disruptions in gut microbiota occur, the loss of microbial diversity and/or overgrowth of potentially pathogenic bacteria can lead to systemic inflammation, immune dysregulation, and impaired barrier functions, all of which negatively affect skin health. Changes to the skin microbiota, which is normally composed of commensal bacteria such as *Staphylococcus epidermidis*, *Cutibacterium acnes*, and *Corynebacterium* spp., can lead to conditions such as acne, eczema, psoriasis, or AD [39].

The gut and skin microbiota interact with the immune system, particularly through the regulation of inflammatory pathways. Gut dysbiosis can result in the release of pro-inflammatory cytokines that travel through the bloodstream to affect distant sites, including the skin. Gut bacteria not only produce SCFAs but also release neurotransmitters (e.g., serotonin) that influence systemic health, including the skin’s immune responses and barrier function [40]. Moreover, the leaky gut phenomenon allows microbial toxins to enter the circulation, leading to systemic inflammation and potentially affecting skin health.

As previously described, in conditions such as acne, dysbiosis can increase inflammation and sebum production while disrupting the balance of skin bacteria and promoting the overgrowth of *C. acnes* [41]. Diets high in refined sugars and dairy can exacerbate this process by influencing insulin and androgen levels, both of which impact the skin. Reduced gut microbial diversity has been associated with a higher risk of eczema, particularly in AD-affected infants [42]. Leaky gut syndrome and AD are both linked to immune dysfunction and inflammation, with emerging evidence suggesting that gut health plays a significant role in the development and exacerbation of AD. This breach could trigger systemic inflammation and overactive immune responses, potentially leading to or worsening autoimmune and inflammatory conditions. The typical Th2-skewed immune response of AD is often linked to allergies, and a leaky gut may amplify this response. Multiple studies supporting the connection between leaky gut and AD suggest that restoring gut health could be a critical strategy in managing AD and other inflammatory skin conditions.

As an example, the worsening of psoriasis has been associated with an altered gut microbiota composition, increased intestinal permeability (leaky gut), and inflammatory signals [43,44]. Gut dysbiosis, particularly the overgrowth of *Helicobacter pylori*, has been implicated in triggering rosacea through systemic inflammation and vascular effects [45]. Conversely, a healthy gut microbiota supports antioxidant production, mitigates oxidative stress, and enhances skin hydration, contributing to delayed aging and improving several inflammatory conditions [3].

### 4.3. Pro-Inflammatory vs. Anti-Inflammatory Foods

A diet rich in pro-inflammatory foods is associated with increased oxidative stress and systemic inflammation, potentially aggravating skin conditions. Conversely, anti-inflammatory foods reduce the frequency and severity of AD flares by modulating inflammation [46]. A high amount of fat and the lack of fermentable fibers are the major determinants for changes in the intestinal microbiota, resulting in increased levels of endotoxin-producing bacteria and pathogen-associated molecular patterns (PAMPs) that act as stimulants for toll-like receptors (TLRs) [47,48]. Indeed, the high consumption of processed food and meat is significantly associated with the high prevalence of AD [49]. In contrast, fermented foods may contain bacteria-associated metabolites that are released during the fermentation process, which are capable of carrying out anti-inflammatory actions through modulation of the gut microbiota [50]. It is known that butyrate, propionate, and other SCFAs produced by the gut microbiota play a pivotal role in different inflammatory diseases, including AD [51]. The Korean National Health and Nutrition Examination Survey, published in 2016, indicates a protective role of fermented foods in AD [49]. Moreover, a study performed on a population of 9763 adults demonstrated that the consumption of traditional Korean fermented food and beer is significantly associated with a lower prevalence of AD. The traditional Korean dish kimchi, consisting of fermented vegetables, contains several *Lactobacillus* strains, some of which ameliorate both AD-like skin lesions and epidermal thickening in mouse models and reduce serum immunoglobulin E (IgE) levels and T-helper cell (Th)-producing cytokines [52]. The beneficial effects of kefir—a fermented food rich in probiotics—have been observed in AD patients, mainly concerning skin hydration [53].

The review published by Khan et al. highlights that exclusive breastfeeding in the first months, prebiotic administration, and a diet rich in fruits and vegetables have a beneficial effect on AD [18]. On the other hand, maternal dietary restrictions during pregnancy and lactation and omega-3 or omega-6 fatty acid supplementation appear to be irrelevant, even if higher prenatal omega-6 levels have been associated with the development of AD in childhood [54]. Probiotics, prebiotics, vitamin D, and unsaturated fats also appear to be beneficial.

Regarding this hot topic, in the three population-based cross-sectional studies by Yajia Li et al., more than 15 thousand Chinese volunteers from different sociocultural and professional contexts were enrolled. Spot urine sample collection for sodium intake analysis, along with a questionnaire and a dermatological assessment, was used to discriminate consumers of processed food from non-consumers based on daily food habits. This was then used to assess people’s health status parameters. What emerged was a strong association between pickles, processed food consumption, and an increase in the risk of developing AD and symptoms. Regarding the association between sodium intake and AD, the results were inconsistent with the methodological approaches used [55]. It is clear that NaCl-mediated signaling can promote the switching of T lymphocytes into the Th2 phenotype, which is an emblematic feature of AD pathogenesis [56,57]. Similarly, Proietti et al. reported an improvement in a single AD patient treated with the anti-IL13/4 antibody dupilumab after conversion from the WD to the MD. In this case, skin amelioration paralleled reductions in weight and fasting blood glucose [58].

### 4.4. Impact of Processed Foods and Additives

**Figure 3:**
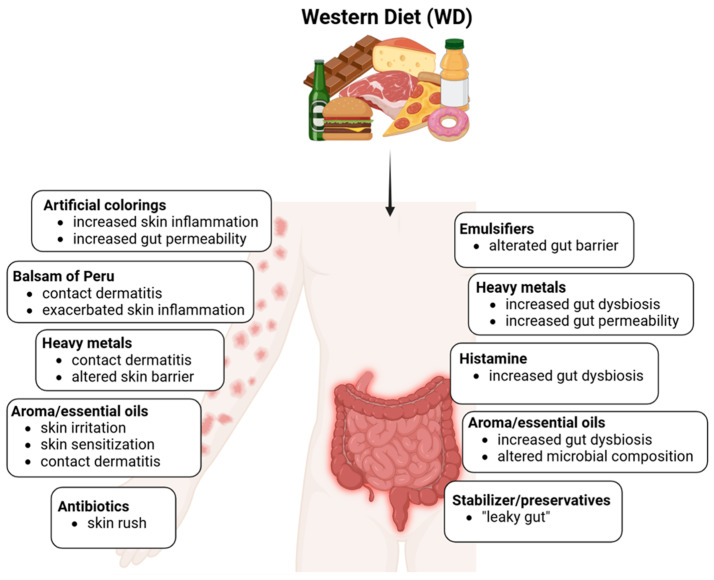
Effects of food additives on the gut and skin barrier and microbiota. Created with Biorender.com.

Processed and ultra-processed food is often rich in additives such as nitrate- and nitrite-based preservatives, colorants, stabilizers, emulsifiers, flavorings or ingredients fermented by bacteria, and histamine. Overall, these components can contribute to and exacerbate the dysbiosis typical of AD [58] (Figure 3).

Preservatives employed by the food industry are designed to extend shelf life and reduce microbial contamination, thus preventing infection risks and foodborne illnesses for the consumer. However, once these additives reach the gastrointestinal niche during digestion, they may act as promoters and accelerators of dysbiosis [59].

As an example, nitrates and nitrites, which are naturally present in both soil and water, are widely used as additives, especially in cured and processed meats, to prevent *Clostridium botulinum* overgrowth and ensure food safety and microbiological preservation [60].

However, growing evidence from several analyses, such as those conducted by the International Agency for Research on Cancer and the NutriNet-Sante cohort study, assesses their procarcinogen potential, which is due to their capacity to significantly increase the risk of colorectal, breast, and prostate cancers [60,61]. They generate *N*-nitroso compounds (NOCs), polycyclic aromatic hydrocarbons (PAHs), and heterocyclic aromatic amines (HAAs), which are enhanced by smoking and high-temperature cooking, and induce DNA mutations. They can trigger a plethora of immune alterations and inflammatory-related diseases and produce reactive oxygen species, which can contribute over time to cancer or fibrosis [60,62].

In this regard, Gonza et al. recently demonstrated how several food additives, including sodium nitrite, can affect the composition of the intestinal microbiota and the metabolic function of both healthy subjects and, to a greater extent, patients affected by intestinal bowel inflammatory disease (IBD), whether they were in remission or not. After incubating the subject’s fecal samples with one preservative at a time in a human intestinal microbial ecosystem (SHIME) simulator, they evaluated microbial α-diversity and load over time [63]. They found that nitrites mainly reduced members of the bacterial *Enterococcus* genus, thus affecting the production of anti-inflammatory metabolites such as SCFAs.

Moreover, nitrates are converted by human microbiota into nitrites, which are associated with an increased risk of cardiovascular diseases, a concern particularly relevant for AD patients, who already face a higher risk compared to the general population.

Further supporting these findings, Willmot et al. perfused an in vitro cultured oral microcosm with nitrate for 7 days and observed an increase in nitrate-reducing taxa such as *Veillonella* and *Neisseria*. These results underscore the intricate interplay between food additives, the microbiota, and systemic health, with significant implications for individuals with chronic inflammatory and cardiovascular conditions [64].

#### 4.4.1. Histamine

As stated by Schnedl and Enko, histamine intolerance originates in the digestive tract due to a genetic or acquired loss or a reduction in diamine oxidase (DAO) activity. Therefore, this results in the incorrect degradation, adsorption, and/or accumulation of this nitrogenous compound [65]. It is related to chemical poisoning, which manifests itself with specific and unspecific intestinal and extraintestinal toxicity, vasomotor and psychotropic symptoms, and systemic pain [66].

Histamine is naturally produced by plants, animals, and human cells, as well as by host microbiota. Not present or present at concentrations lower than 10 μg/g of fresh food, this biogenic amine can reach higher levels (above 50 μg/g) in mature cheese, bacterium- or yeast-fermented foods, and processed fish, meat, or eggs. Unfortunately, a WD has increased food-chain histamine levels, as well as dysbiosis-associated pathologies, thus reducing host enzyme production or saturating their processing capabilities.

Moreover, subjects with respiratory and cardiac pathologies, gastrointestinal disorders associated with hypertension, and vitamin B6, B12, and C deficiency, as well as those who take drugs that inhibit these enzymes, are more at risk as they are more sensitive to small quantities of amines. This is because the activity of oxidases in their bowel is generally lower than in healthy individuals [67].

High histamine levels have also been retrieved in both plasma and inflamed skin in AD. This organic pleiotropic molecule is a pivotal player in the disease’s chronic symptoms through the activity of its four transmembrane G protein-coupled receptors named H1, H2, H3, and H4 [68,69]. Recent research in mouse and human models shows how antagonistic drugs against the H4 receptor have strong anti-inflammatory and pruritic effects much greater than those against the H1–H3 receptors [70].

Low-histamine diets have been proposed to counteract the symptoms of histamine intolerance [71,72]. Depending on their histamine content, certain foods should be avoided in order not to trigger any clinical manifestations. However, this approach still has some limitations, namely the lack of consensus on foods that must be excluded from the patient’s dietary regimen, despite the growing supporting evidence [73].

Moreover, histamine is not the only toxic biogenic amine. Among others, tyramine should be considered as it has previously been shown to possibly cause allergic reactions in a small cohort of pediatric AD patients after a food additive challenge test [74].

#### 4.4.2. Emulsifiers

Food emulsifiers are defined by Codex Alimentarius as additives that form or maintain a uniform emulsion of two or more phases within a food [75]. Their structure is both hydrophilic and hydrophobic, allowing them to reduce the surface tension between the oil and water phases and thus prevent oil droplets from separating from the mixture. In their absence, phenomena such as creaming, sedimentation, flocculation, coalescence, or parting can occur [76]. Several authors such as Sozener et al. highlight that exposure to environmental substances such as food emulsifiers can compromise the epithelial barriers of the skin and respiratory and gastrointestinal tracts, increasing vulnerability to allergens and external microbes [17]. In this regard, the study by Chassaing et al. demonstrates that food emulsifiers such as polysorbate 80 (P-80) and carboxymethylcellulose (CMC) directly alter the composition and gene expression of the human gut microbiota, increasing its pro-inflammatory potential, as evidenced by the increase in bioactive flagellin and transfers of modified microbiota in mouse models [77]. Oscarsson et al. highlight that P-80 and sodium CMC can also increase intestinal permeability, promoting systemic inflammation. This leaky gut could contribute to increased immune activation that extends from the intestinal mucosa to the skin, worsening AD symptoms. While further studies are needed to confirm the observed data, these findings suggest that emulsifiers could be an aggravating factor for inflammatory diseases such as AD [78]. The recent study by Naimi et al. also confirms the effects of emulsifiers on intestinal health revealed by other researchers. The authors observed that exposure to P-80 and sodium CMC significantly alters the composition of the intestinal microbiota, favoring the growth and virulence of opportunistic pathogenic bacteria such as *Clostridium difficile* and *Escherichia coli,* compromising the intestinal barrier [79].

#### 4.4.3. Stabilizers and Preservatives

The impact of food additives on both skin and gut microbiota is a subject of increasing study as they influence microbial communities within the body. Stabilizers, used to maintain the consistency of food products, can interact with the gut microbiota by modifying the intestinal environment, potentially leading to microbiota imbalance that may further affect immune and metabolic functions [80]. Recent research showed that stabilizers and additives in processed foods can disturb the gut microbiota, leading to alterations in species composition by promoting the growth of certain bacterial groups over others and possibly contributing to conditions such as obesity and metabolic syndromes [80].

The study by Worm et al. examined the role of food stabilizers in adult AD patients, highlighting that preservatives may contribute to symptom aggravation in some sensitized individuals. Substances such as sulfites and benzoates, commonly used to preserve food, are found to be among the main causes of adverse reactions, including the worsening of skin inflammation and itching. Since such responses were not seen in all participants, the study suggests that preservatives represent a potential trigger in a subpopulation of patients [81]. Zaknun et al. also examined their role, highlighting their ability to trigger inflammatory responses and compromise the immune system in predisposed individuals. Sulfites, for example, can increase systemic inflammation through the modulation of oxidative pathways, potentially aggravating chronic inflammatory conditions such as AD. Furthermore, preservative-induced intestinal epithelial barrier dysfunction could promote allergic sensitization, a cascade of immune events that include the worsening of skin symptoms. Although a direct link to AD requires further investigation, preservatives emerge as possible aggravating factors in this context [82].

#### 4.4.4. Colorings

The impact of artificial colorings on gut and skin microbiota has been increasingly investigated as they are widely used in processed foods and can influence health through microbiome interactions. Studies suggest that artificial food colorings, particularly synthetic azo dyes such as Allura Red (E129) and tartrazine (E102), can disrupt the gut microbial environment. These dyes are thought to increase gut permeability and alter the balance of gut flora, potentially leading to dysbiosis, inflammatory disorders, and systemic inflammation, which may indirectly impact skin health. In the case of azo dyes, studies have observed that such synthetic compounds can promote the growth of pathogenic bacteria or inhibit beneficial strains, such as *Lactobacillus* and *Bifidobacterium* genera, which are crucial for gut health [83].

The study by Van Bever et al. was pioneering in exploring the role of artificial colorings in severe AD. Among the dyes examined, E129 and E102 were identified as potential aggravators, with effects mediated by immunological (e.g., histamine release following non-IgE-mediated reactions) or non-immunological mechanisms. The study highlights the importance of recognizing these sensitivities through elimination diets and oral challenge tests, paving the way for a more targeted approach in AD management [74]. The results of the study by Worm et al. highlighted that these food colorings can also aggravate the adverse reactions of some AD patients. Through oral provocation tests, it has been observed that these additives can intensify itching and skin inflammation in predisposed subjects [81]. According to the study by Zaknun et al., E129 and E102 can contribute to allergic and inflammatory reactions, with potentially relevant implications for AD. They increase oxidative stress and stimulate an overactive immune response, factors that can aggravate systemic inflammatory processes [82]. This finding underscores the broader implications of diet, including color additives, on skin conditions through gut microbiota modulation. Avoiding synthetic colorants and supporting microbiota with a diet rich in fiber and prebiotics can be a practical approach to maintaining both gut and skin health.

#### 4.4.5. Aromatic and Essential Oils

The impact of aromatic compounds, specifically essential oils (EOs) and plant-based aromas, on the skin and gut microbiota has garnered attention for their potential influence on microbial communities. These compounds are often used in the food industry, pharmaceutical products, and cosmetic formulations [84,85,86]. Although still in an embryonic stage, research on these compounds shows how they can have truly different effects in each subject depending on their microbial heritage. EOs contain bioactive compounds such as terpenes, phenols, and aldehydes that can influence the composition and functionality of microbial communities in the body.

Lavender, thyme, and sweet orange EOs have shown antimicrobial and anti-inflammatory effects that are generally positive for the gut by promoting the growth of beneficial bacteria such as *Lactobacillus* spp. while inhibiting pathogenic strains such as *E. coli* and *Clostridium perfringens*. Nevertheless, their effects can vary significantly, depending on the composition and concentration of their active volatile and penetrating compounds, as well as their persistence. Some EOs, such as high-dose thymol or carvacrol, may disrupt the microbial balance, potentially leading to dysbiosis, which underscores the need for extreme caution to avoid adverse effects that are not easily controlled. Further research is needed to fully understand the individual dosage and long-term implications of aromatic compounds on microbiota health across different populations and settings.

From a dermatological point of view, conflicting data can be found. On the one hand, lavender EO from *Lavandula angustifolia* exerts an AD-protective effect by inhibiting AhR activation in an in vitro AD cell model. Moreover, they do not show any skin-sensitization potential [87]. Other studies highlight the anti-inflammatory and anti-AD activities of *Mentha arvensis* EO through the inhibition of the ERK/NF-κB signaling pathway and NLRP3 inflammasome activation in animal models and in vitro cell models [88,89]. Despite these results, it should be noted that EOs have shown toxicity, especially through oral administration [90], and are contact sensitizers as they contain many compounds (e.g., cinnamic aldehyde and phenylacetaldehyde) with allergic potential. The sensitization reactions may be different if known sensitizers are used individually or combined, so great attention should be paid to fragrance-based products [91]. Aromatherapy has been associated with adverse dermatological reactions, such as dermatitis on the hands, among its practitioners, while skin irritation and contact dermatitis have been reported as the most common adverse effects [90].

#### 4.4.6. Heavy Metals

Heavy metals, including lead, cadmium, mercury, and arsenic, can enter the body through ingestion, inhalation, or direct skin contact and can disrupt the microbial balance by generating oxidative stress, altering epithelial barrier functions, and promoting inflammation [92,93].

Indeed, they have a significant impact on the gut microbiota, with major local and systemic consequences, such as affecting the metabolic profile and brain homeostasis via the gut–brain axis [94,95]. Data regarding their direct effects on skin microbiota are still lacking, despite the growing evidence of skin dysbiosis induced by environmental pollutants [96].

In the gut, heavy metal-associated dysbiosis may contribute to health issues such as IBD, obesity, and metabolic syndrome [97,98]. Gut commensals have different susceptibilities to heavy metals and detoxification capabilities. However, heavy metals can deplete beneficial gut symbionts, resulting in a loss of healthy local biodiversity. For instance, exposure to arsenic leads to an increase in arsenic-resistant genera and to the horizontal transfer of determinants of both antibiotic and metal resistance [99,100]. Heavy metals also dramatically shape the metabolic profile of the gut microflora (e.g., SCFA production and the profile of simple amino acids), therefore altering the host’s metabolic health [101,102].

On the skin, heavy metals similarly disrupt the epithelial barrier. As demonstrated in vitro by Chavatte et al., lead and nickel can penetrate the epidermis and deposit in the dermal layer, associating with local oxidative phenomena and genotoxic consequences [103]. Exposure, most notably to nickel, is responsible for contact dermatitis and extracellular matrix weakening via the overexpression of matrix-degrading enzymes (e.g., metalloproteinase 2) [104]. Pro-inflammatory pathways are activated as well, establishing a feedback loop with detrimental consequences [103,104,105].

It is believed that skin commensals may be involved in host sensitization to heavy metals since both the microorganisms and the metals trigger the same immunological pathways. Shang et al. reported that co-exposure of reconstructed human skin to nickel and *Streptococcus mitis*, an oral and cutaneous human commensal, strongly enhances pathways involved in innate immunity, especially CXCL8 secretion [106]. Co-exposure to cadmium and diethylhexyl phthalate, a commonly used plasticizer, on a *Rana chinensis* model by Jiang et al. showed their different impacts on the microbiota and health of animal skin with respect to the untreated control [107].

Focusing on AD, prenatal and/or early life exposure to several heavy metals is under investigation, especially regarding AD development in children. Ho et al. highlighted how prenatal exposure to nickel has immunological effects on children at age 3, as they reported a significant association between maternal nickel exposure and decreased AD risk [108]. The MOCEH cohort study in South Korea reported an association between cord blood cadmium levels and the odds of developing AD [109]. Similar results were recorded in the EDEN birth cohort in France, as it reported that a greater risk of developing eczema was associated with high cord blood levels of cadmium and high mid-pregnancy maternal manganese levels [110].

#### 4.4.7. Balsam of Peru

Balsam of Peru (Table 2), derived from the resin of *Myroxylon pereirae*, is known for its antimicrobial, anti-inflammatory, and potential skin-healing properties. It has been widely used in topical applications for various skin issues, including eczema and minor wounds. Its complex mix of natural compounds can offer antimicrobial benefits, which help to protect the skin by inhibiting the growth of harmful bacteria on its surface. Balsam of Peru, as a complete blend, has been increasingly replaced by individual constituents or fractions that are now used in foods, confectionery, baked goods, chocolate, confectionery, and medicinal ointments. Its ingredients can also be added to tobacco [111].

In terms of impact on the microbiota, balsam of Peru’s antimicrobial action may affect the balance of skin and respiratory flora; it is a known allergen and can cause contact dermatitis, especially in those with sensitive skin and other conditions. The study by Herro et al. analyzed a clinical case of systemic contact dermatitis in a sample of seven children sensitized to balsam of Peru. In particular, the consumption of ketchup, containing spices and ingredients derived from balsam of Peru, caused widespread skin reactions typical of systemic contact dermatitis. Symptoms include inflammation that is not limited to areas of direct contact but also extends to other body regions. The study highlighted that the elimination of foods containing balsam of Peru from the diet leads to a clear clinical improvement [112].

De Groot et al. analyzed the possible link between the presence of balsam of Peru in foods and AD, highlighting that the ingestion of foods containing derivatives of this resin, such as spices, citrus fruits, chocolate, and flavored drinks, can aggravate skin symptoms in a subpopulation of sensitized patients. Although some patients with AD may benefit from eliminating foods containing balsam of Peru, the author urges caution when adopting generalized restrictive diets, recommending a personalized approach [113,114].

**Table 2:** Ingredients and composition (%) identified in Myroxylon pereirae resin (MP), extracts, and essential oils [113].

INGREDIENT	COMPOSITION (%)	INGREDIENT	COMPOSITION (%)
Amyrin		α-Farnesene and β-farnesene	
Aristolene		Farnesol	Traces
Benzaldehyde		Ferulic acid	0.1–0.4%
Benzoic acid	1.5–11%	Formic acid	
Benzyl alcohol	1–2%	Geranyl acetone	
Benzyl benzoate	up to 30%	Guaiacol	
Benzyl cinnamate	up to 40%	Heptadecanoic acid (margaric acid)	
Benzyl p-coumarate (benzyl-*trans*-4-hydroxycinnamate)		Hexacosanoic acid (cerotic acid)	
Benzyl ferulate		1-Hexacosanol	
Benzyl formate		Hexadecanoic acid (palmitic acid)	
Benzyl isoferulate (*cis* and *trans*)	0.2%	Hydroconiferyl benzoate	
Benzyl vanillate (benzyl 4-hydroxy-3-methoxybenzoate)		Hydroconiferyl cinnamate	
*cis*-α-Bisabolene, β-bisabolene and cis-γ-bisabolene and trans-γ-bisabolene		Hydroxycinnamic acid	
β-Caryophyllene		Isoeugenol	0.85% in fraction BP3
1,8-Cineole		Isoferulic acid (traces)	
*cis*-Cinnamic acid and *trans*-cinnamic acid	3–30%	Lactic acid (2-hydroxypropanoic acid)	
Cinnamyl alcohol	0.4%	Limonene	
Cinnamyl cinnamate	0.5%	Methoxyeugenol	
Coniferyl alcohol	0.2%	Methyl benzoate	
*cis*-Coniferyl benzoate and trans-coniferyl benzoate	up to 1.5% in fresh MP	Methyl cinnamate	
Coniferyl cinnamate		Methyl vanillyl ketone	
α-Copaene		α-Muurolene	
α-Curcumene		Naphthalene	
Cycloisosativene		Nerolidol	2–7%
p-β-Cymene and trans-β-cymene		allo-β-Ocimene, *cis*-β-ocimene and *trans*-β-ocimene	
Docosanoic acid		1-Octacosanol	
Dodecanoic acid		Patchoulene	
Eicosanoic acid (arachidic acid)		α-Phellandrene and β-phellandrene	
Ethylbenzene		1-Phenylethanol (α-methylbenzyl alcohol)	
Ethyl benzoate		3-Phenylpropanol	
Ethyl cinnamate		α-Pinene and β-pinene	
Ethylhexanoic acid (tentatively identified)		β-Sesquiphellandrene	
Eugenol	0.2% in fraction BP3	Stearic acid (octadecanoic acid)	
Styrene		Tetradecanoic acid (myristic acid)	
α-Terpinene and γ-terpinene		1-Undecanol	
4-Terpineol (terpinen-4-ol)		Vanillic acid (4-hydroxy-3-methoxybenzoic acid)	
α-Terpineol		Vanillin	0.2–1.3%
1-Tetracosanol (lignoceryl alcohol)		p-Vinylguaiacol	

#### 4.4.8. Antibiotics

The extensive use of antibiotics in agriculture and livestock farming represents a serious threat to public health, as highlighted by the study by Chang et al. These drugs, used with the aim of guaranteeing animal growth and preventing or resolving infectious diseases, encourage the selection of resistant bacteria that spread to humans through contaminated meat, water, soil, and other environmental routes. Such transfers of resistance genes not only reduce the effectiveness of antibiotics in clinical settings and deplete the reserve of the individual human microbiota, generating serious and persistent dysbiosis, but also make individuals more susceptible to exogenous and endogenous infections, complicating their treatment [115]. The study by Baynes et al. highlights the risks associated with antibiotic residues in foods of animal origin, including the possibility of skin reactions such as dermatitis or urticaria. Some antibiotics, such as penicillin, sulfonamides, and tetracyclines, which are known to trigger allergic or hypersensitive reactions, can contaminate food and generate systemic inflammation in sensitive consumers, with effects ranging from mild skin irritation to anaphylactic shock [116]. The study by Bacanlı et al. confirms how antibiotic residues present in foods of animal origin can pose a risk to human health, including the development of adverse reactions such as skin irritation. The previously mentioned penicillin and tetracyclin can leave traces in animal-derived food products and trigger allergic reactions such as rashes, itching, or contact dermatitis after exposure [117].

### 4.5. Nutritional Deficiencies and Skin Barrier Function

Diets lacking in essential fatty acids (EFAs), vitamins (especially A, C, D, and E), and minerals such as zinc and selenium can significantly impair the skin barrier, making it more susceptible to dryness, irritation, and inflammation. In AD, where it is already compromised, they can exacerbate the condition by allowing easier penetration of allergens and irritants [118].

EFAs, particularly omega-3 and omega-6 fatty acids, are crucial components of the skin’s lipid barrier and are involved in the production of ceramides and other lipids that maintain skin hydration and integrity. For instance, dietary deficiency of linoleic acid has been shown to cause AD-like lesions in mice [118]. Supplementation with EFAs, such as gamma-linolenic acid (GLA) found in evening primrose oil and borage oil, has been reported to improve skin barrier function and reduce AD symptoms [119]. However, a review of 12 clinical trials on the use of borage oil for AD showed variable results, with significant clinical effects observed only in some studies [120].

Other studies suggest that vitamin A and vitamin D deficiencies are associated with increased AD severity [121,122,123]. Vitamin A is essential for skin cell growth and differentiation and helps maintain structural integrity, while its deficiency can lead to hyperkeratosis and impaired barrier function, increasing susceptibility to irritation [124]. Vitamin D plays a role in skin immune function and has anti-inflammatory properties; its supplementation may alleviate symptoms due to its immunomodulatory effects, although clinical trials have shown mixed results [125].

An inadequate intake of vitamin C has been observed in AD patients [126]. Vitamin C protects the skin from oxidative stress and is necessary for collagen synthesis, which is vital for skin elasticity and strength. While vitamin C supplementation may have promising positive effects in AD patients, further research is needed [127].

Vitamin E acts as a powerful antioxidant, supporting skin barrier function and exerting anti-inflammatory effects that are useful in reducing the severity of AD symptoms. Vitamin E deficiency has been considered in AD. Low plasmatic vitamin E concentrations were found in animal models and vitamin E supplementation has been suggested as an adjunctive AD treatment [128,129]. In addition, the Mendelian randomization analysis recently performed by Wang et al. suggested a causal relationship between vitamin E intake and AD manifestations; on the contrary, the same study failed to demonstrate a possible causative role of vitamins A and C [130].

Vitamins are not only introduced through dietary habits. K and B vitamins are also synthesized by vitamin prototroph bacteria in the large intestine [131]. These microbial-derived vitamins support both the host and other auxotrophic commensals, with several pairs of bacteria that metabolically complement each other [132,133]. It has been suggested that microbial dysbiosis and vitamin deficiency may be connected and could have a notable impact on the host’s health. Several studies focused on microbial dysbiosis, vitamin deficiency, and metabolic diseases, such as Crohn’s disorder and type 2 diabetes, but more investigation into their connection with AD is needed [134]. For instance, Chesini and Caminati suggested an association between vitamin B12 blood levels and AD severity or relapse. In their case report, AD flares corresponded with hematic vitamin B12 deficiency. After oral supplementation, normal levels of vitamin B12 were recorded, as well as improved SCORAD index values [135]. Although this study did not investigate the involvement of gut microbiota, it could represent a starting point for future research.

As far as minerals are concerned, zinc is crucial for skin repair and regeneration, possessing anti-inflammatory and antioxidant properties that aid in reducing skin inflammation and promoting healing in AD. Prolonged zinc deficiency is associated with a Th2-skewed immune response, the maturation of macrophages and dendritic cells (DCs), and the generation of IgE antibodies [136]. In contrast, adequate zinc levels establish immune resilience, promote regulatory cells, and induce tolerance. Clinical trials have shown that zinc supplementation can reduce inflammation in various skin disorders, including AD [137,138].

### 4.6. Antioxidants and Skin Protection

Diets rich in antioxidants, which are found in food such as berries, green tea, and dark chocolate, play a significant role in protecting skin cells from oxidative stress, a key contributor to skin aging and inflammation. By reducing free radical damage and enhancing the body’s natural defense mechanisms, antioxidants offer benefits for inflammatory skin conditions such as AD. Furthermore, a high intake of antioxidants may neutralize some of the harmful effects of environmental pollutants, protecting the skin barrier from external insults that could aggravate inflammatory responses [139].

In studies involving hairless mice, oral administration of β-carotene significantly reduced AD-like skin inflammation by suppressing pro-inflammatory protein expression and enhancing filaggrin expression [140]. Similarly, antioxidant compounds such as β-carotene and lycopene have been shown to prevent the onset of AD-like dermatitis in mice by suppressing Th2 activity [141].

Several cross-sectional studies conducted in different countries have demonstrated that the consumption of fruit and vegetables exerts a protective effect against atopic diseases in children [142]. However, a review published by Fsadni et al. highlighted numerous biases and inconsistencies in dietary evaluation methods across various studies, underscoring the need for more validated assessment tools [143].

The benefits of a plant-based diet for inflammatory skin diseases have also been explored. This type of diet is rich in food with antioxidant properties and is characterized by a low glycemic load, which has a favorable impact on metabolic syndrome and inflammatory skin conditions. A study published by Kouda et al. evaluated the effects of a low-energy plant-based diet in AD patients, demonstrating a significant reduction in the SCORAD index after 8 weeks [144]. All participants also experienced weight loss and reductions in BMI and systolic blood pressure. A plant-based dietary regimen is enriched in fiber and bioactive compounds that can improve gut dysbiosis, potentially contributing to its beneficial effects on skin health.

## 5. Maternal Diet and AD

Even if the role of dietary restriction during pregnancy and lactation needs to be clarified, several papers in the literature suggest a putative role of maternal nutrition in AD pathogenesis. A diet rich in antioxidants during pregnancy has been associated with a reduced risk of developing AD and other atopic conditions in the offspring, even among those with a genetic predisposition [145]. Moreover, vitamins A, C, D, and E, copper, zinc, and magnesium, as well as the consumption of vegetables, fish, and dairy, demonstrated a protective role against atopic outcomes in children. In 2022, a Polish study conducted on 557 mother–child pairs demonstrated that the inadequate intake of vitamin E during pregnancy is significantly associated with a higher risk of AD [146]. In addition, maternal supplementation of fish oil has been associated with a lower risk of allergic sensitization to food in childhood [147]. An inadequate magnesium intake is associated with a high risk of wheezing, whereas, surprisingly, WD and unhealthy diet regimens were associated with a higher risk of infections but a lower AD risk [146].

In addition, alcohol consumption before pregnancy was associated with a risk of developing atopic eczema in the first three years of life [148]. Since the maternal diet can also impact the risk of atopy through the modulation of microbiota, the role of probiotics may be interesting. Recent evidence supports the hypothesis that maternal supplementation during pregnancy may reduce the risk of AD [147].

## 6. Pitfalls and Future Possibilities

Despite mounting evidence supporting the pivotal role of diet in AD, several critical gaps remain. Many studies lack standardized dietary assessments, complicating efforts to pinpoint specific nutrients, contaminants, or additives that most significantly influence skin dysbiosis and inflammatory pathways. Furthermore, the complex interaction between genetic predisposition and environmental factors, including diet, is not yet fully elucidated, limiting the development of personalized treatment strategies. To address these challenges, robust longitudinal studies and well-controlled clinical trials are urgently needed to clarify the mechanisms by which dietary factors modulate immune responses and the composition of gut/skin microbiota. Advancing our understanding of these dynamics could pave the way for tailored nutritional interventions, offering more precise and effective preventive and therapeutic options for managing AD within the broader exposome framework.

## 7. Conclusions

In summary, the exposome, and more specifically diet, plays a complex and multifactorial role in AD, modulating the immune response, skin barrier integrity, and microbiota. Understanding and managing exposome factors is essential for developing targeted preventive and therapeutic strategies, improving the quality of life of AD patients.

## Data Availability

Not applicable.
